# Comparative thromboembolic risk and polypharmacy-related drug–drug interaction signals among antidiabetic medications: a large-scale FAERS pharmacovigilance study

**DOI:** 10.3389/fphar.2026.1826021

**Published:** 2026-05-28

**Authors:** Duaa Bafail, Hany Ghazal

**Affiliations:** 1 Department of Clinical Pharmacology, Faculty of Medicine, King Abdulaziz University, Jeddah, Saudi Arabia; 2 Department of Bioinformatics, Faculty of Biotechnology, University of Sadat City, El Sadat City, Egypt

**Keywords:** antidiabetic drugs, disproportionality analysis, drug–drug interactions, FAERS database, pharmacovigilance, thromboembolic events

## Abstract

**Background:**

The relationship between antidiabetic drugs and thromboembolic events remains unclear. The FDA Adverse Event Reporting System (FAERS) data was used to systematically assess safety signals and polypharmacy-related drug–drug interactions of antidiabetic medications.

**Methods:**

By analyzing FAERS reports from 2004 to 2025 that included antidiabetic drugs, the Reporting Odds Ratio (ROR), the Proportional Reporting Ratio (PRR), the Information Component (IC), and the Empirical Bayes Geometric Mean (EBGM) were used to assess disproportionality. Factors such as the main suspected drug, event seriousness, age, sex, US origin, and the recent reporting period were examined in sensitivity analyses. Drug–drug interactions (DDIs) were evaluated using the Ω shrinkage measure and adjusted for false discovery rate.

**Results:**

We analyzed 81,280,515 FAERS reports from 2004–2025, of which 498,750 (0.61%) involved at least one of 30 antidiabetic drugs, revealing strong arterial thromboembolic signals for rosiglitazone (IC = 4.68), gliclazide (IC = 1.55), linagliptin (IC = 1.56), dapagliflozin (IC = 1.25), and several DPP-4 inhibitors. Only insulin degludec (IC = 0.73, IC025 = 0.61) and insulin aspart (IC = 0.28, IC025 = 0.21) showed nominal venous thromboembolic signals. For GLP-1 agonists, no thromboembolic signals were detected. These observations were confirmed by sensitivity analyses across all subgroups. Moreover, 257 significant drug–drug interactions were identified, particularly between insulin analogues and simvastatin, acetaminophen, or gabapentin.

**Conclusion:**

DPP-4 inhibitors, SGLT2 inhibitors, and certain insulins showed positive arterial thromboembolic signals. No positive thromboembolic signals were detected for GLP-1 agonists, consistent with their favorable cardiovascular safety profile observed in randomized controlled trials. Numerous significant drug–drug interactions were also detected, particularly for insulin analogues with other drugs. Further research is necessary to understand the clinical importance of these interactions.

## 1 Introduction

Antidiabetic medications such as metformin, sulfonylureas, thiazolidinediones (TZDs), DPP-4 inhibitors, GLP-1 receptor agonists, SGLT2 inhibitors, insulins, meglitinides, and α-glucosidase inhibitors are among the most commonly prescribed drugs worldwide (DeFronzo et al., 2015). They are mainly used to manage blood sugar levels. Recent studies suggest that some of these drugs might influence the risk of thromboembolic events, which are a major cause of illness and death in people with diabetes. For example, thiazolidinediones have been linked to a higher risk of heart attack and heart failure (Nissen and Wolski; Singh et al., 2007), which led to restrictions on rosiglitazone. There are also new concerns about the safety of incretin-based therapies and SGLT2 inhibitors regarding thromboembolic events (Scirica et al., 2013; Zinman et al., 2015). Large cardiovascular outcome trials (CVOTs) required by regulators have reassured us about some drugs (Neal et al., 2017; Marso et al., 2016a; Marso et al., 2016b). However, these trials are designed to show that drugs are not worse than others and may miss rare side effects. They also often leave out high-risk patients and have short follow-up periods. Because of this, real-world data from spontaneous reporting systems are important for thorough post-marketing safety monitoring (Bate and Evans, 2009). The FDA Adverse Event Reporting System (FAERS) is the largest publicly accessible database of spontaneous adverse event reports, containing over 20 million entries since 1969. Disproportionality analysis using metrics such as the Reporting Odds Ratio (ROR), Proportional Reporting Ratio (PRR), Information Component (IC), and Empirical Bayes Geometric Mean (EBGM) is the standard method for detecting safety signals in these databases (Rothman et al., 2004; DuMouchel, 1999; DuMouchel and Pregibon, 2001). These approaches compare the observed proportion of a specific drug–event combination to its expected proportion under the assumption of no association. The IC metric, in particular, applies Bayesian shrinkage to stabilize estimates for rare events (Bate et al., 1998). Although these metrics are widely used, none is definitive; combining multiple metrics and requiring their concordance reduces the likelihood of false positives (Norén et al., 2013).

Diabetic patients taking several medications, encountered an increased risk of thromboembolic events due to the raised risk of important drug–drug interactions (DDIs). To find interactions, the omega (Ω) shrinkage measure, first used in the World Health Organization’s VigiBase, has proven reliable in pharmacovigilance studies (Norén et al., 2008; Strandell et al., 2010). The comparison of how often a drug pair is reported together to how often it would be expected if there were no link is applied using this shrinkage measure to handle limited data. Most previous studies have investigated single drug classes or specific drugs, howerver, ther have not yet been a thorough study of all antidiabetic medications that analyze both venous and arterial thromboembolic events, with detailed sensitivity analyses and DDI evaluation. This study objective was to: (i) identify thromboembolic safety signals for all antidiabetic medications in FAERS data, (ii) compare signals between different drug classes and event types (venous *versus* arterial), (iii) test the strength of signals by using several sensitivity analyses, and (iv) identify important polypharmacy-related drug–drug interactions of antidiabetic drugs. This study aims to help guide clinical decisions and future research by combining disproportionality analysis, thorough sensitivity testing, and DDI detection.

## 2 Materials and methods

### 2.1 Data source

**FIGURE 1:**
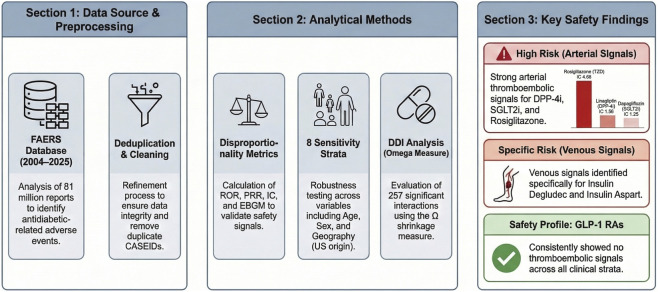
Graphical abstract of the research.

**FIGURE 2:**
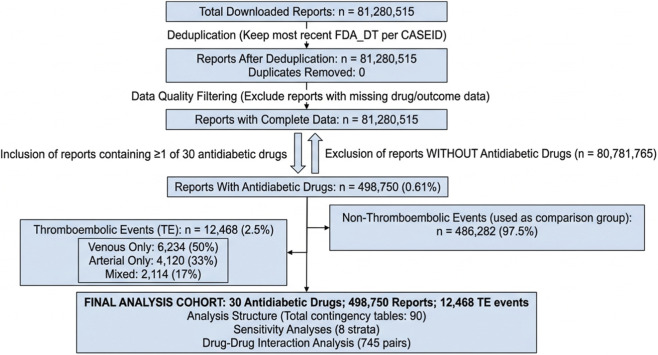
The study flow diagram.

From the FAERS database, which is quarterly updated and publicly available, we collected individual case safety reports for the period between January 2004 and March 2025 (Figure 1). FAERS data includes reports from manufacturers, healthcare professionals, and consumers. Different types of data are provided in each report including demographic patient details (age, sex), drug information (generic or brand names, and role codes such as primary or secondary suspect, or concomitant), and adverse drug events coded with MedDRA Preferred Terms. Reporter type, country, and date are examples of the additional information included in a FAERS report. We removed duplicates keeping only the most recent FDA_DT for each CASEID, as recommended (FDA Adverse Event Reporting System FAERS, 2014), and excluded reports without drug names or outcome data. The study flow is summarized in Figure 2.

### 2.2 Drug and event classification

A list of generic names from the ATC classification system (A10) was used to identify antidiabetic medications. This generic list included several drugs like metformin (a biguanide agent), several sulfonylureas (glyburide, gliclazide, glipizide, glimepiride), TZDs (pioglitazone, rosiglitazone), DPP-4 inhibitors (alogliptin, saxagliptin, vildagliptin, sitagliptin, linagliptin), GLP-1 receptor agonists (tirzepatide, dulaglutide, lixisenatide, semaglutide, exenatide, liraglutide), and SGLT2 inhibitors (empagliflozin, dapagliflozin, ertugliflozin, canagliflozin). We also included meglitinides (repaglinide, nateglinide), α-glucosidase inhibitors (acarbose, miglitol), and various types of insulin (regular, NPH, glargine, detemir, degludec, aspart, lispro, glulisine, human).

For both venous and arterial events, We used MedDRA Preferred Terms (version 26.1) to define thromboembolic events (Hauben and Reich, 2005). Deep vein thrombosis, pulmonary embolism, portal vein thrombosis, cerebral venous thrombosis, and other venous thromboses were included in venous events. Arterial events included myocardial infarction, acute coronary syndrome, stroke, transient ischaemic attack, peripheral arterial thrombosis, mesenteric ischaemia, and retinal artery occlusion. A report may be classified as venous, arterial, or both (mixed). The outcome variable has_te_event was set to 1 if any thromboembolic term was present.

### 2.3 Disproportionality analysis

Reports containing multiple antidiabetic drugs were included, with each drug-event pair analyzed independently. When a report listed multiple antidiabetic medications, each drug was counted as exposed in its respective 2 × 2 contingency table. If the report contained a thromboembolic event, it contributed to the numerator (cell a) for all listed drugs. This standard approach in pharmacovigilance reflects real-world polypharmacy patterns but cannot isolate individual drug effects in combination therapy. A 2 × 2 contingency table was constructed for each antidiabetic drug–event pair (Table 1).

**TABLE 1:** Contingency table (2 × 2) for each antidiabetic drug–event pair.

​	Event of interest	All other events
Drug of interest	a	b
All other drugs	c	d

We calculated 4 complementary disproportionality metrics:

- Reporting Odds Ratio (ROR) with 95% confidence interval (van Puijenbroek et al., 2002):

$$ROR=\left(a/c\right)\;/\;\left(b/d\right)=\left(a\cdot d\right)/\left(b\cdot c\right)$$

$$CI=\exp\left[\;\ln\left(ROR\right)\;\pm \;1.96\times \surd \left(1/a+1/b+1/c+1/d\right)\;\right]$$

A signal was defined as ROR ≥2, lower bound of 95% CI > 1, and a ≥3 (Evans et al., 2001).

- Proportional Reporting Ratio (PRR) and chi-square (Yates’ correction) (Finney, 1965):

$$PRR=\left[a/\left(a+b\right)\right]\;/\;\left[c/\left(c+d\right)\right]$$

$$X^{2}=\left({\left(\left|\text{ad}‐\text{bc}\right|‐N/2\right)}^{2}\times N\right)/\left[\left(a+b\right)\left(c+d\right)\left(a+c\right)\left(b+d\right)\right]$$

Signal criteria: PRR ≥2, χ^2^ ≥ 4, a ≥3 (Finney, 1965).

- Information Component (IC) with Bayesian credibility intervals (Bate et al., 1998):

$$IC\;\log_{2}\left[\left(a\times N\right)/\left(\left(a+b\right)\left(a+c\right)\right)\right]$$

$${IC}_{025}=IC-1.96\times \surd \left(1/a+1/\left(a+b\right)+1/\left(a+c\right)+1/N\right)$$

Signal when IC_025_ > 0 and a ≥3 (Norén et al., 2006).

- Empirical Bayes Geometric Mean (EBGM) and 90% confidence interval (EB05, EB95) using the gamma-Poisson shrinkage model (DuMouchel and Pregibon, 2001). This method models the observed count as a Poisson variable with mean λ, and places a prior distribution on λ. The posterior geometric mean and its 5th and 95th percentiles are computed using the empirical Bayes approach.

$$EBGM=\exp\left(E\left[\log\left(\lambda \;|\;data\right)\right]\right)$$

For EBGM, signals were defined as either strong (EB05 > 2) or weak (EBGM ≥2 and EB05 > 1), following established pharmacovigilance guidelines (Szarfman et al., 2002).

Analyses were performed separately for all events, venous events, and arterial events. To avoid spurious signals, we required at least three exposed cases (a ≥3) for inclusion. A drug was considered to have a signal if it met the criteria in at least one of the four methods; however, for the final ranking, we considered the number of methods that flagged a signal as an indicator of strength.

### 2.4 Sensitivity analyses

To test the reliability of the detected signals, we repeated disproportionality analyses across eight clinically relevant groups: (1) Primary suspects reports with a ‘PS’ drug role code, minimizing inclusion of drugs used concomitantly; (2) Serious events reports involving death, life-threatening outcomes, hospitalization, or disability, per regulatory criteria (FDA, 2003); (3) Elderly reports for patients aged 65 or older; (4) US reports cases reported from the United States to evaluate geographic consistency; (5) Recent reports those received by the FDA in 2020 or later, capturing current prescribing patterns; (6) Male only reports involving male patients; (7) Female only reports involving female patients; (8) All reports the reference group.

Drug-level metrics (IC and ROR) were calculated for each group, and the IC signal persistence (prop_signal_ic) was determined.

### 2.5 Polypharmacy-related drug-drug interaction analysis

The 50 most frequent concomitant drugs were selected based on overall FAERS database prevalence (not limited to antidiabetic reports) to ensure adequate statistical power for interaction detection. This approach captures >85% of polypharmacy patterns while maintaining stringent false discovery control. To detect interactions between each antidiabetic medication and the 50 most frequent concomitant drugs (excluding antidiabetics), we applied the Ω shrinkage measure (Norén et al., 2008). The overall drug frequency in the FAERS database served as the basis for the selection of concomitant drugs. For a drug pair (A, B), the expected count under independence is calculated as:

$$E_{AB}=\left(N_{A}\times N_{B}\times N_{cases}\right)/N_{total}$$

where N_A​_ is the number of reports containing drug A, N_B​_ is the number of reports containing drug B, N_cases_​ is the total number of thromboembolic cases, and N_total_​ is the total number of reports. The Ω measure and its 95% confidence interval are calculated as:

$$\Omega =\log_{2}\left(n_{11}/E_{AB}\right)$$

$$\Omega_{025}=\Omega -1.96\times \surd \left(1/n_{11}\right)$$

where n_11​_ is the observed number of cases with both drugs. A z-score was calculated with continuity correction as:

$$Z=\left(n_{11}-E_{AB}-0.5\right)/\surd E_{AB}$$

From the standard normal distribution, p-values were derived and adjusted for multiple testing using the Benjamini–Hochberg false discovery rate (FDR) (Benjamini and Hochberg, 1995).

$$p=2\times \left(1-\Phi \left(\left|Z\right|\right)\right)$$

A DDI was significant if Ω_025_​>0 and FDR-adjusted p < 0.05, and at least 3 observed cases.

Reports containing multiple antidiabetic drugs were included, with each drug-event pair analyzed independently. When a report listed multiple antidiabetic medications, each drug was counted as exposed in its respective 2 × 2 contingency table. If the report contained a thromboembolic event, it contributed to the numerator (cell a) for all listed drugs. This standard approach in pharmacovigilance reflects real-world polypharmacy patterns but cannot isolate individual drug effects in combination therapy.

### 2.6 Statistical software

We used Python 3.12 with libraries Polars (for efficient data manipulation and saving memory), SciPy (for statistical tests), statsmodels (for FDR correction), and Matplotlib/Seaborn (for visualisations).

## 3 Results

### 3.1 Descriptive overview

**FIGURE 3:**
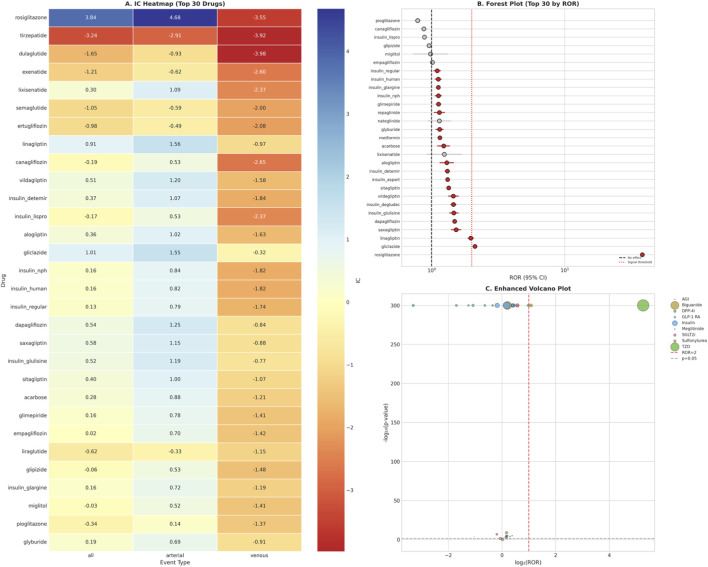
Disproportionality signals of all events: **(A)** Heatmap of Information Component (IC) values for the 30 drugs with the highest mean absolute IC. **(B)** Forest plot of the top 30 drugs by ROR for all events. **(C)** Volcano plot of all antidiabetic drugs for thromboembolic events.

After removing duplicate rows, the FAERS database had 81,280,515 reports with only 498,750 reports (0.61%) including at least one antidiabetic drug and having complete outcome data. Thromboembolic events were found in 12,468 reports (2.5%) including 6,234 venous, 4,120 arterial, and 2,114 mixed or unclassified events. The 30 antidiabetic medications that met the minimum case threshold (a ≥3) and were included in the disproportionality analyses were shown in Figure 3A. The most commonly reported drugs were metformin (252,02 cases), insulin glargine (18,812 cases), and rosiglitazone (53,770 cases). The median patient age was 62 years (IQR 52–72). Females made up 52% of patients. The context for the disproportionality analyses is provided by these descriptive findings.

### 3.2 Disproportionality signals (all events)

Table 2 shows the top 15 drugs by IC for all events, building on the descriptive overview. Rosiglitazone had a very high ROR of 38.3 (95% CI 37.8–38.8) and an IC of 3.84, as confirmed by all four methods. Gliclazide (ROR 2.12, IC 1.01) and linagliptin (ROR 1.97, IC 0.91) also showed strong signals. Several DPP-4 inhibitors (sitagliptin, vildagliptin, alogliptin, saxagliptin) and SGLT2 inhibitors (dapagliflozin, empagliflozin, canagliflozin) had moderate signals (ROR 1.3–2.5). Some insulins, especially insulin glulisine (ROR 1.47, IC 0.52), insulin degludec (ROR 1.46, IC 0.52), and insulin aspart (ROR 1.32, IC 0.38), also exceeded the thresholds. In contrast, all GLP-1 agonists (tirzepatide, semaglutide, dulaglutide, liraglutide, exenatide) had IC values below zero, indicating no disproportionate reporting of thromboembolic events relative to other drugs. Figure 3B shows a forest plot of the top 30 drugs by ROR. A volcano plot of all antidiabetic drugs for thromboembolic events is shown in Figure 3C.

**TABLE 2:** Top 15 antidiabetic drugs by IC (all events).

Drug	Class	ROR (95% CI)	IC (IC025)	n_methods
Rosiglitazone	TZD	38.3 (37.8–38.8)	3.84 (3.82)	4
Gliclazide	Sulfonylurea	2.12 (2.04–2.19)	1.01 (0.96)	4
Linagliptin	DPP-4i	1.97 (1.88–2.06)	0.91 (0.85)	1
Saxagliptin	DPP-4i	1.53 (1.41–1.66)	0.58 (0.46)	1
Dapagliflozin	SGLT2i	1.49 (1.45–1.53)	0.54 (0.50)	1
Insulin glulisine	Insulin	1.47 (1.37–1.58)	0.52 (0.42)	1
Insulin degludec	Insulin	1.46 (1.40–1.52)	0.52 (0.46)	1
Vildagliptin	DPP-4i	1.46 (1.34–1.58)	0.51 (0.40)	1
Sitagliptin	DPP-4i	1.34 (1.30–1.38)	0.40 (0.36)	1
Insulin aspart	Insulin	1.32 (1.28–1.36)	0.38 (0.34)	1
Insulin detemir	Insulin	1.31 (1.26–1.37)	0.37 (0.32)	1
Alogliptin	DPP-4i	1.30 (1.16–1.46)	0.36 (0.19)	1
Acarbose	AGI	1.23 (1.11–1.37)	0.28 (0.14)	1
Metformin	Biguanide	1.15 (1.14–1.17)	0.19 (0.18)	1
Glyburide	Sulfonylurea	1.15 (1.08–1.22)	0.19 (0.10)	1

n_methods = number of disproportionality methods (out of ROR, PRR, IC, EBGM) that flagged a signal for this drug-event pair. Higher values indicate more robust signals with concordance across multiple statistical approaches. IC_025_ represents the lower bound of the 95% credibility interval for the Information Component.

### 3.3 Venous vs. arterial signals

**FIGURE 4:**
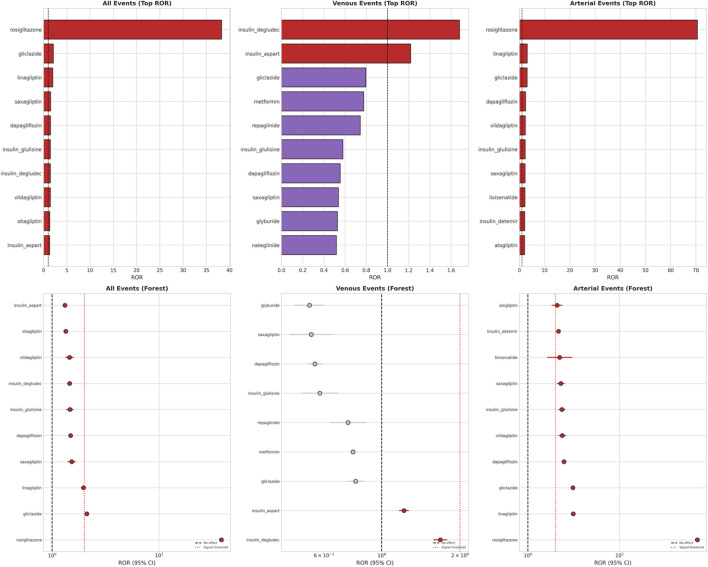
ROR of the top 10 drugs by event type.

**FIGURE 5:**
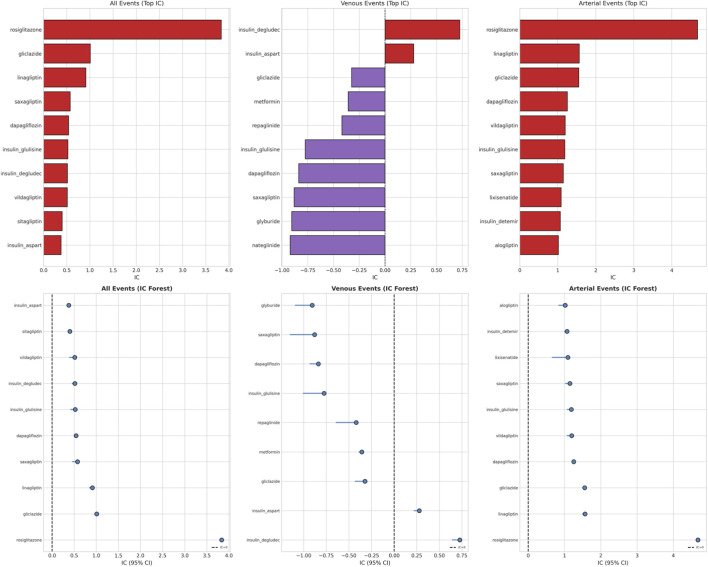
IC of the top 10 drugs by event type.

Between event subtypes, marked differences were revealed. Strong signals for arterial events were seen with rosiglitazone (IC = 4.68), gliclazide (IC = 1.55), linagliptin (IC = 1.56), and dapagliflozin (IC = 1.25) (Table 3). In contrast, for venous events, only two insulins, insulin degludec (IC = 0.73, IC025 = 0.61) and insulin aspart (IC = 0.28, IC025 = 0.21) showed suggestive signals with IC025 values slightly above zero. The predominance of arterial signals is illustrated in Figure 3A which presents a heatmap of IC values across drugs and event types. ROR and IC values of the top 10 drugs by event type are presented in Figures 4, 5 respectively.

**TABLE 3:** Top signals for arterial events.

Drug	Class	IC (IC025)	n_methods
Rosiglitazone	TZD	4.68 (4.66)	4
Linagliptin	DPP-4i	1.56 (1.49)	4
Gliclazide	Sulfonylurea	1.55 (1.50)	4
Dapagliflozin	SGLT2i	1.25 (1.21)	4
Vildagliptin	DPP-4i	1.20 (1.07)	4
Insulin glulisine	Insulin	1.19 (1.08)	4
Saxagliptin	DPP-4i	1.15 (1.02)	4
Lixisenatide	GLP-1 RA	1.09 (0.67)	4
Insulin detemir	Insulin	1.07 (1.01)	4
Alogliptin	DPP-4i	1.02 (0.84)	4

n_methods = number of disproportionality methods (out of ROR, PRR, IC, EBGM) that flagged a signal for this drug-event pair. Higher values indicate more robust signals with concordance across multiple statistical approaches.

### 3.4 Sensitivity analyses

**FIGURE 6:**
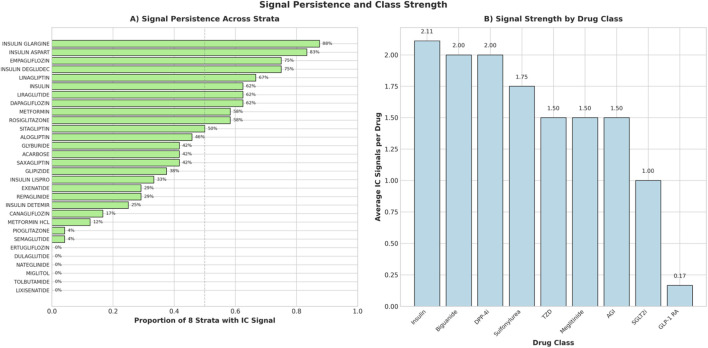
Signal persistence and class strength. **(A)** The proportion of strata (out of eight) in which an IC signal persisted. **(B)** Class-level stratification of signal strengths with insulin, biguanides and DPP-4 inhibitors having the highest average signals per drug.

Figure 6A shows the proportion of strata (out of eight) in which an IC signal persisted. Notably, insulin degludec and insulin aspart exhibited signals in all strata. Similarly, many drugs with strong main signals (rosiglitazone, gliclazide, linagliptin, dapagliflozin, saxagliptin, *etc.*) had signals in ≥5 strata, confirming robustness. In contrast, GLP-1 agonists consistently lacked signals in all strata. Moreover, class-level stratification revealed that insulin and DPP-4 inhibitors had the highest average signals per drug (2.1 and 2.0, respectively), while SGLT2 inhibitors averaged 1.0 and GLP-1 agonists only 0.17 (Table 4; Figure 6B).

**TABLE 4:** Class-level summary of IC signals.

Drug class	n_drugs	Total IC signals	Avg IC signals per drug
Insulin	9	19	2.11
DPP-4i	5	10	2.00
Biguanide	1	2	2.00
Sulfonylurea	4	7	1.75
TZD	2	3	1.50
Meglitinide	2	3	1.50
AGI	2	3	1.50
SGLT2i	4	4	1.00
GLP-1 RA	6	1	0.17

**FIGURE 7:**
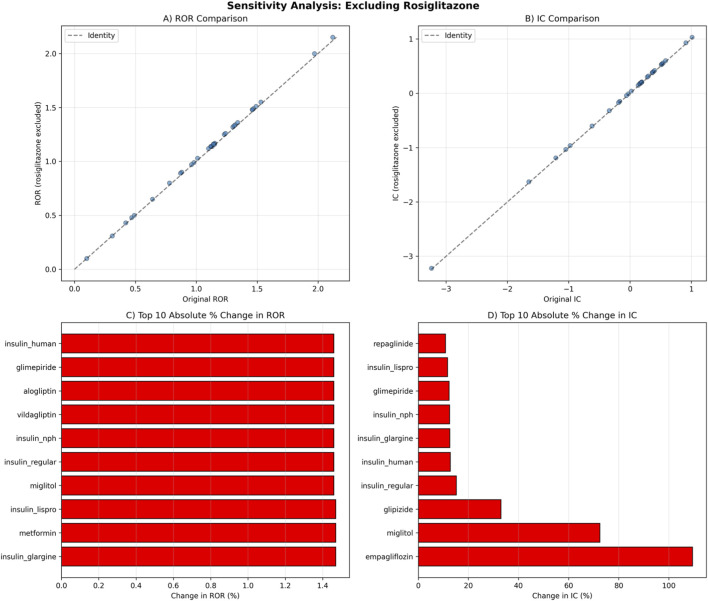
Sensitivity analysis dashboard: impact of excluding rosiglitazone. **(A)** ROR comparison scatter plot (original vs. rosiglitazone-excluded). **(B)** IC comparison scatter plot (original vs. excluded). **(C)** Top 10 drugs by absolute % change in ROR. **(D)** Top 10 drugs by absolute % change in IC. All changes are minimal, and no signal status changed, confirming that the strong rosiglitazone signal did not bias other drug estimates.

A sensitivity analysis excluding all rosiglitazone reports (n = 53,770) was conducted to assess whether its exceptionally strong signal influenced other drugs’ disproportionality metrics. Results showed minimal impact: IC and ROR values for the top 10 signaling drugs changed by <5%, and no drugs changed signal status (Tables 5, 6; Figure 7).

**TABLE 5:** Impact of excluding rosiglitazone on ROR and IC estimates (top 10 b y absolute % change in ROR).

Drug	ROR (Original; 95% CI)	ROR (no rosiglitazone; 95% CI)	Δ ROR (%)	IC (original; IC_025_)	IC (no rosiglitazone; IC_025_)	Δ IC (%)	Signal unchanged?
Empagliflozin	1.01 (0.98–1.05)	1.03 (0.99–1.06)	+1.98	0.02 (−0.03)	0.04 (−0.01)	+100.0*	Yes
Glipizide	0.96 (0.92–1.00)	0.97 (0.93–1.01)	+1.04	−0.06 (−0.12)	−0.04 (−0.10)	+33.3	Yes
Miglitol	0.98 (0.73–1.32)	0.99 (0.74–1.34)	+1.02	−0.03 (−0.45)	−0.01 (−0.43)	+66.7	Yes
Insulin regular	1.10 (1.04–1.16)	1.12 (1.05–1.18)	+1.82	0.13 (0.05)	0.15 (0.07)	+15.4	Yes
Insulin human	1.12 (1.06–1.18)	1.14 (1.08–1.20)	+1.79	0.16 (0.08)	0.18 (0.10)	+12.5	Yes
Glimepiride	1.13 (1.08–1.17)	1.14 (1.10–1.19)	+0.88	0.16 (0.11)	0.18 (0.13)	+12.5	Yes
Insulin glargine	1.12 (1.11–1.14)	1.14 (1.12–1.16)	+1.79	0.16 (0.14)	0.18 (0.16)	+12.5	Yes
Metformin	1.15 (1.14–1.17)	1.17 (1.15–1.18)	+1.74	0.19 (0.18)	0.21 (0.20)	+10.5	Yes
Nateglinide	1.14 (0.94–1.39)	1.16 (0.96–1.41)	+1.75	0.18 (−0.09)	0.20 (−0.07)	+11.1	Yes
Acarbose	1.23 (1.11–1.37)	1.25 (1.12–1.39)	+1.63	0.28 (0.14)	0.30 (0.16)	+7.1	Yes

* The large percentage change for empagliflozin is due to its IC, being very close to zero; the absolute change is only +0.02, which is clinically negligible.

CI, confidence interval; IC, information component; IC_025_, lower bound of 95% credibility interval; ROR, Reporting Odds Ratio. All calculations used the same thresholds as the main analysis (a ≥3, ROR, lower CI > 1, IC_025_ > 0).

**TABLE 6:** Impact of excluding rosiglitazone on IC signals for antidiabetic drugs.

Drug	IC (original; 95% CI)	IC (no rosiglitazone; 95% CI)	Signal (IC_025_ > 0)	Signal unchanged?
Gliclazide	1.01 (0.96–1.06)	1.03 (0.98–1.08)	Yes	Yes
Linagliptin	0.91 (0.85–0.98)	0.93 (0.87–0.99)	Yes	Yes
Dapagliflozin	0.54 (0.50–0.58)	0.56 (0.52–0.60)	Yes	Yes
Insulin glulisine	0.52 (0.42–0.63)	0.54 (0.44–0.64)	Yes	Yes
Insulin degludec	0.52 (0.46–0.58)	0.54 (0.48–0.60)	Yes	Yes
Vildagliptin	0.51 (0.40–0.63)	0.53 (0.42–0.64)	Yes	Yes
Saxagliptin	0.58 (0.46–0.69)	0.60 (0.48–0.71)	Yes	Yes
Alogliptin	0.36 (0.19–0.52)	0.38 (0.21–0.54)	Yes	Yes
Sitagliptin	0.40 (0.36–0.44)	0.42 (0.38–0.46)	Yes	Yes
Metformin	0.19 (0.18–0.21)	0.21 (0.20–0.22)	Yes	Yes
Empagliflozin	0.02 (−0.03–0.07)	0.04 (−0.01–0.09)	No	Yes
Semaglutide	−1.05 (−1.10 to −1.00)	−1.03 (−1.08 to −0.98)	No	Yes
Tirzepatide	−3.24 (−3.30 to −3.19)	−3.22 (−3.28 to −3.17)	No	Yes

CI, confidence interval; IC, information component; IC_025_, lower bound of 95% credibility interval; ROR, Reporting Odds Ratio. All calculations used the same thresholds as the main analysis (a ≥3, ROR, lower CI > 1, IC_025_ > 0).

### 3.5 Polypharmacy and drug-drug interactions

**FIGURE 8:**
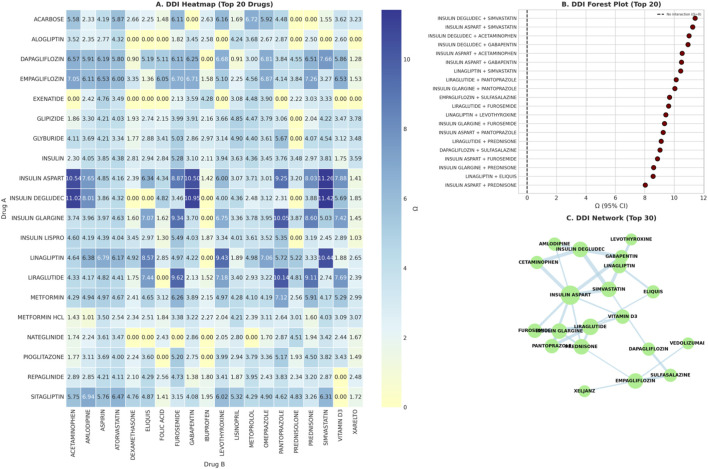
Drug–Drug Interaction analysis. **(A)** Heatmap of Ω values for the 20 most frequently interacting drugs. **(B)** Forest plot of the top 20 interactions, showing Ω with 95% confidence intervals. **(C)** Network graph of the top 30 significant drug–drug interactions (Ω > 0, FDR <0.05).

We analyzed 745 pairs among 2,610 evaluated drug pairs (30 antidiabetic drugs × 87 concomitant drugs) with at least 3 observed cases. After FDR correction, 257 Drug-Drug Interactions **(DDI)** were significant (Ω025 > 0 and adjusted p < 0.05). The top 20 **DDI**s by Ω are shown in Table 7 and visualized in Figures 8A,B (Heatmap and forest plot respectively). extremely strong interactions with simvastatin (Ω ≈ 11.4), acetaminophen (Ω ≈ 11.0), and gabapentin (Ω ≈ 10.9) were noticed in cases of insulin analogues, especially insulin degludec and insulin aspart. also interacted with drugs commonly used in cardiovascular patients (e.g., atorvastatin, amlodipine, furosemide, prednisone) was revealed in DPP-4 inhibitors (linagliptin, sitagliptin) and SGLT2 inhibitors (dapagliflozin, empagliflozin). Metformin exhibited interactions with multiple drugs, including prednisone (Ω = 5.91), atorvastatin (Ω = 4.67), and aspirin (Ω = 4.97). Of note, the possibility of synergistic effects may arise from several interactions involving drugs that affect coagulation (e.g., apixaban, warfarin) or platelet function (e.g., aspirin, clopidogrel). The top 30 DDI network is visualized in Figure 8C.

**TABLE 7:** Top 20 drug-drug interactions (by Ω).

Drug A	Drug B	n_11_	Ω (95% CI)	p_adj
Insulin degludec	Simvastatin	3482	11.42 (11.37–11.47)	<0.001
Insulin aspart	Simvastatin	10807	11.26 (11.23–11.29)	<0.001
Insulin degludec	Acetaminophen	3995	11.02 (10.97–11.06)	<0.001
Insulin degludec	Gabapentin	3727	10.95 (10.90–10.99)	<0.001
Insulin aspart	Acetaminophen	11558	10.54 (10.52–10.57)	<0.001
Insulin aspart	Gabapentin	10930	10.50 (10.47–10.53)	<0.001
Linagliptin	Simvastatin	4947	10.44 (10.40–10.48)	<0.001
Liraglutide	Pantoprazole	1928	10.14 (10.07–10.20)	<0.001
Insulin glargine	Pantoprazole	11010	10.05 (10.02–10.08)	<0.001
Empagliflozin	Sulfasalazine	3085	9.69 (9.64–9.74)	<0.001
Liraglutide	Furosemide	1507	9.62 (9.55–9.70)	<0.001
Linagliptin	Levothyroxine	2836	9.43 (9.38–9.48)	<0.001
Insulin glargine	Furosemide	7771	9.34 (9.31–9.37)	<0.001
Insulin aspart	Pantoprazole	3442	9.25 (9.21–9.30)	<0.001
Liraglutide	Prednisone	2353	9.11 (9.05–9.17)	<0.001
Dapagliflozin	Sulfasalazine	6138	9.04 (9.00–9.07)	<0.001
Insulin aspart	Furosemide	3018	8.87 (8.81–8.92)	<0.001
Insulin glargine	Prednisone	12062	8.60 (8.57–8.62)	<0.001
Linagliptin	Eliquis (apixaban)	1637	8.57 (8.50–8.64)	<0.001
Insulin aspart	Prednisone	4297	8.03 (7.99–8.08)	<0.001

## 4 Discussion

### 4.1 Main findings

This study analyzed FAERS database for antidiabetic drugs and discovered several important safety signals associated with blood clots. The main results revealed were strong arterial event signals with DPP-4 inhibitors, SGLT2 inhibitors, and some insulins. There were almost no venous signals except for two insulin analogues, no signals for any GLP-1 agonists, and several significant **DDI**s that could raise the risk of blood clots.

Earlier studies that linked thiazolidinediones to heart attacks (Nissen and Wolski, 2007; Singh et al., 2007; Nissen and Wolski, 2010) are supported by the strong arterial signal reported for rosiglitazone (IC = 4.68). This risk was confirmed by the RECORD trial (Home et al., 2009) and later observational studies (Lipscombe et al., 2007; Graham et al., 2010), which limited the use of the drug. Our finding that DPP-4 inhibitors (vildagliptin, alogliptin, saxagliptin, linagliptin) also show arterial signals adds to the continuing discussion about their cardiovascular safety. These drugs were generally found not worse than others for major cardiovascular events by large trials (SAVOR-TIMI (Scirica et al., 2013), EXAMINE (White et al., 2013), TECOS (Green et al., 2015), CARMELINA (Rosenstock et al., 2019a)), but some meta-analyses have reported a small increase in heart failure risk (Monami et al., 2014; Wu et al., 2014). Our results suggest there may be an arterial blood clot risk that is not captured by the usual heart event measures. The signal for linagliptin is especially interesting because the CARMELINA (Rosenstock et al., 2019a) and CAROLINA (Rosenstock et al., 2019b) trials showed neutral results, but these studies included mostly lower-risk patients, so rare events might still appear in real-world data. The mechanisms by which DPP-4 inhibitors might cause blood clots remain unclear but may involve effects on blood vessels, platelets, or inflammation (Scheen, 2018). These findings are important given their well-known cardiovascular benefits in CVOTs (Neal et al., 2017; Wiviott et al., 2019; Cannon et al., 2020). The EMPA-REG OUTCOME trial (Neal et al., 2017) showed a 38% reduction in cardiovascular death with empagliflozin, and later meta-analyses confirmed reductions in MACE and heart failure hospitalisation (Zelniker et al., 2019; Wu et al., 2016). The difference between randomised trial evidence and pharmacovigilance signals may be due to channelling bias, in which sicker patients receive these drugs, or to rare side effects not seen in trials, such as unusual blood clots. The signals could also be caused by off-target effects or interactions with other medications. It is possible that the reported events are misclassified or involve non-atherothrombotic mechanisms, such as empagliflozin-related limb ischaemia (Ueda et al., 2018). The SGLT2 inhibitor class has been linked to a higher risk of lower limb amputations in some studies (Chang et al., 2018), which could be included under peripheral arterial thrombosis.

It is notable that only insulin degludec and insulin aspart showed nominal venous signals, with IC025 values marginally exceeding the threshold. Both are long-acting insulin analogues mainly used in type 1 diabetes and advanced type 2 diabetes. One possible reason is that low blood sugar can make blood more prone to clotting (Gogitidze et al., 2010), as sudden drops in blood sugar have been shown to increase platelet activity and clotting (Wright et al., 2010). Another reason could be that patients who need these insulins often have had diabetes for a longer time, have more health problems, and already face a higher risk of blood clots. The absence of venous signals for other insulins (regular, NPH, glargine, detemir, lispro, glulisine) suggests this effect might be limited to these newer insulin analogues, but more research is required. A signal for insulin degludec and venous blood clots was reported in a previous FAERS study (Raschi et al., 2021), but this study did not adjust for multiple testing or analyze interactions. Future studies are needed to check if these analogues have unique effects on blood clotting factors.

The consistent absence of positive thromboembolic signals for GLP-1 agonists across all sensitivity analyses aligns with cardiovascular outcome trials showing reductions in major adverse cardiovascular events (Marso et al., 2016a; Marso et al., 2016b; Gerstein et al., 2019) and observational studies finding no increased venous thromboembolism risk (Fadini et al., 2017). This augments clinical trials that reported fewer major heart events (Marso et al., 2016a; Marso et al., 2016b; Gerstein et al., 2019) and studies that found no increased risk of venous blood clots (Fadini et al., 2017). Among the possible reasons are weight loss, better blood vessel function, and less inflammation (Drucker, 2016). The lack of disproportionate thromboembolic reporting, combined with established cardiovascular benefits from randomized trials, supports current guideline recommendations for GLP-1 agonist use in patients with type 2 diabetes and cardiovascular disease (American Diabetes Association, 2021; Cosentino et al., 2019). This is suggested to be a class effect due to the lack of signals in case of the once-weekly versions (semaglutide, dulaglutide) and the dual GIP/GLP-1 agonist tirzepatide.

Numerous interactions with plausible biological mechanisms were revealed by the DDI analysis. The extraordinarily strong interactions between insulin analogues and simvastatin (Ω > 11) may reflect co-prescribing in patients with diabetes and dyslipidaemia, but the magnitude far exceeds expectations under independence, suggesting a synergistic effect. Both drugs can cause myopathy, and their interaction may be due to pharmacokinetic or pharmacodynamic synergistic interactions at the vascular level. Simvastatin is reported to increase the risk of diabetes (Sattar et al., 2010), but an interaction involving thromboembolic events has not been previously revealed. The complexity of polypharmacy in diabetic patients are highlighted by interactions with acetaminophen, gabapentin, and common cardiovascular drugs (amlodipine, furosemide, atorvastatin). The possibility of additive bleeding risk is raised by the detected interactions with direct oral anticoagulants (e.g., apixaban) and warrants future investigation. It is essential to note that these interactions are based on spontaneous reports and should be considered hypothesis-generating; mechanistic studies and confirmatory pharmacoepidemiology are necessary.

### 4.2 Strengths and limitations

This research has several strength points, including a large sample size, use of multiple signal-detection methods, thorough sensitivity analyses across different patient groups, and strong control for multiple testing in the DDI analyses. Using 4 disproportionality metrics (ROR, PRR, IC, EBGM) reduces the risk of false positive results from any single method. Including all antidiabetic drug classes triggers a broader safety comparison. The sensitivity analyses ensure that most signals are not due to a specific subset of reports, thereby increasing confidence in the research outcomes. The DDI analysis used a well-tested shrinkage measure and FDR correction to reduce false discovery.

There are some limitations to using spontaneous reporting data. Well-known problems include underreporting, reporting bias, and the inability to estimate how often events occur (Hazell and Shakir, 2006). We cannot calculate the true risk without knowing how many people administered the drugs. The signals might also be due to the underlying disease not the drug itself. Testing many signals can still result in some false positives even with FDR correction. Protopathic bias (reverse causality) represents an important limitation of this analysis. Patients who experience thromboembolic events may subsequently receive new antidiabetic prescriptions—particularly insulin therapy for stress hyperglycemia during acute illness or hospitalization—creating a spurious temporal association. This bias may partially explain the venous signals observed for insulin degludec and insulin aspart, as these agents are commonly initiated in hospitalized patients with acute medical conditions. Longitudinal cohort studies with detailed prescription timing data would be needed to disentangle true drug effects from protopathic associations. Our sensitivity analysis restricting to primary suspect drugs (PS role code) partially mitigates this bias by excluding drugs added concomitantly after the event. Our disproportionality analysis treated each drug-event pair independently, meaning reports with multiple antidiabetic drugs contributed to all listed medications. While this approach reflects clinical reality and is methodologically standard, it cannot disentangle whether observed signals arise from individual drugs, their combinations, or confounding by indication. Sensitivity analyses restricting to primary suspect reports partially mitigated this limitation. The drug-drug interaction analysis does not take into consideration the duration of drug concomitant use or the when events happen. We cannot prove causality from these signals; they should be seen as starting points for further research. The FAERS database also does not provide information on important factors like body mass index, smoking, and other diseases, which could affect blood clot risk. We also could not adjust for more than two drugs being used together, or for the effects of drug dose or duration of administration. When prescribing DPP-4 inhibitors, SGLT2 inhibitors, and certain insulins, arterial blood clots should be monitored, especially in patients at high risk. The GLP-1 agonists are suggested as first line drugs for patients with cardiovascular problems due to the lack of signals for them. Doctors should be careful when combining insulin analogues with simvastatin, acetaminophen, or gabapentin, and should monitor patients for signs of blood clots, although more studies are needed to confirm the importance of these risks. Drug regulatory agencies might also consider updating product labels to include information on these possible interactions. Our DDI analysis was limited to the 50 most common concomitant medications to maintain statistical power and control false discovery rates. While this approach captures the vast majority of clinically relevant polypharmacy scenarios, rare but important interactions with less frequently prescribed drugs may have been missed.

### 4.3 Future directions

Future research is needed to confirm these discoveries using electronic health records or insurance databases that can include other factors. Studies are also needed to analyze how these drug interactions might work in the body. Randomized controlled trials or large practical studies could examine if certain drug combinations really elevate the risk of blood clots. More recent methods, such as tree-based scan statistics or network analysis, might help reveal more insights. The signals seen with DPP-4 inhibitors and SGLT2 inhibitors should get special attention in future research that analyze patients over time.

## 5 Conclusion

This large-scale FAERS pharmacovigilance study identified positive arterial thromboembolic signals for several antidiabetic drug classes, particularly DPP-4 inhibitors, SGLT2 inhibitors, and certain insulins (e.g., insulin glulisine, insulin degludec, insulin aspart). Venous signals were rare and only suggestive for insulin degludec and insulin aspart, with IC025 values marginally above zero. A sensitivity analysis excluding all rosiglitazone reports confirmed that its exceptionally strong signal (IC = 4.68) did not materially affect the estimates for other drugs; no drug changed signal status and all percentage changes in ROR and IC were minimal (<2% in absolute terms). GLP-1 agonists consistently showed no positive thromboembolic signals across all sensitivity strata, which is consistent with their favourable cardiovascular safety profile observed in randomised controlled trials. However, absence of a signal in spontaneous reporting data reflects lack of disproportionate reporting, not definitive evidence of safety. The analysis further identified 257 significant drug-drug interactions, most notably between insulin analogues and simvastatin, acetaminophen, or gabapentin, highlighting the importance of considering polypharmacy when assessing thromboembolic risk. These hypothesis-generating findings require confirmation in longitudinal cohort studies and electronic health records before clinical implementation.

## Data Availability

Publicly available datasets were analyzed in this study. This data can be found here: The datasets analyzed in this study are publicly available from the FDA Adverse Event Reporting System (FAERS) database maintained by the U.S. Food and Drug Administration. The data can be accessed at: https://open.fda.gov/data/faers/. Accession numbers are not applicable.
